# AAV-Based Gene Therapy: Opportunities, Risks, and Scale-Up Strategies

**DOI:** 10.3390/ijms26178282

**Published:** 2025-08-26

**Authors:** Daniil Moldavskii, Zarema Gilazieva, Alisa Fattakhova, Valeriya Solovyeva, Shaza Issa, Albert Sufianov, Galina Sufianova, Albert Rizvanov

**Affiliations:** 1 Institute of Fundamental Medicine and Biology, Kazan Federal University, Kazan 420008, Russia; daniilmoldavskiy2@gmail.com (D.M.); zegilazieva@gmail.com (Z.G.); alisashajmardanova@kpfu.ru (A.F.); vavsoloveva@kpfu.ru (V.S.); 2 Department of Genetics and Biotechnology, St. Petersburg State University, St. Petersburg 199034, Russia; shaza.issa98@outlook.com; 3 Department of Neurosurgery, Sechenov First Moscow State Medical University of the Ministry of Health of the Russian Federation (Sechenov University), Moscow 119991, Russia; sufianov_a_a@staff.sechenov.ru; 4 The Research and Educational Institute of Neurosurgery, Peoples’ Friendship University of Russia (RUDN), Moscow 117198, Russia; 5 Department of Pharmacology, Tyumen State Medical University, Tyumen 625023, Russia; sufarm@mail.ru; 6 Division of Medical and Biological Sciences, Tatarstan Academy of Sciences, Kazan 420111, Russia

**Keywords:** gene therapy, AAV production, gene therapy vectors, high-titer AAV, AAV manufacturing

## Abstract

Currently, the development of adeno-associated virus (AAV)-based gene therapy is a promising method for treating various diseases and is gaining increasing popularity. However, the use of AAV has certain drawbacks and faces limitations such as immune responses and an increased risk of insertional mutagenesis, which have not always been adequately considered in the context of AAV therapy. Moreover, a significant limitation for the application of AAV lies in the challenge of producing it in large quantities. This article discusses the use of AAV in treating various diseases, reviews AAV production approaches, highlights challenges with insufficient viral titers during production, and explores potential solutions at key stages of AAV drug production.

## 1. Introduction

Currently, the treatment of genetic disorders remains a pressing issue. Estimates indicate that approximately 2–3% of all newborns are affected by such conditions. There are more than 7000 different genetic disorders [1]. The combined prevalence of inherited metabolic disorders—monogenic diseases—is 1/800 [2]. It is important to note that accurate statistics for these types of diseases are primarily maintained in the most developed countries. For instance, according to statistics, 10% of the U.S. population suffers from genetic disorders, but effective treatments are available for only 5% of these individuals [3]. According to the EURORDIS organization, about 30 million patients with rare genetic diseases are registered in the European Union. In the Russian Federation, about 17,500 patients have been registered with 24 life-threatening and chronically progressive rare diseases [4].

Genetic disorders result from mutations that can lead to severe and difficult-to-treat pathologies. Disease-causing genetic information can reside in nuclear and mitochondrial DNA, as well as in the microbiome and its metagenome, suggesting that alterations in the surface proteins of microbial cells may contribute to the development of pathological conditions [5].

Conventional pharmaceutical methods currently offer only symptomatic treatment. In contrast, gene therapy enables the correction of genetic mutations by replacing the mutated genomic region, introducing a functional copy of the affected gene or employing genome editing technologies [5]. Between 2015 and 2020, the most frequently treated diseases using gene therapy included hemophilia A, Duchenne muscular dystrophy, spinal muscular atrophy, and sickle cell anemia [6]. In addition to these conditions, several drugs have been approved by the U.S. Food and Drug Administration (FDA) for the treatment of spinal muscular atrophy, Leber’s congenital amaurosis [7], and other disorders (Table 1).

There are four main approaches to gene therapy: gene addition, gene silencing, gene replacement, and gene editing [8]. These are implemented either through genome editing using clustered regularly interspaced short palindromic repeats (CRISPR) or via vector-based delivery (both viral and non-viral) [8]. Currently, non-viral delivery systems fail to meet many therapeutic requirements, requiring significant effort due to their complex composition, with each individual drug needing to be developed from scratch [9]. As for viral delivery systems, several vectors are currently being studied: retroviruses, lentiviruses, adenoviruses, herpesviruses, and AAVs (Table 2) [10].

Among the existing options for gene therapy, recombinant AAV-based vectors are the most promising and widely used in therapy. They offer several clinical advantages. AAVs can transduce non-dividing cells while maintaining the same level of gene expression as in actively dividing cells [23]. Furthermore, AAVs have various serotypes with tropism for specific tissues and organs, as well as the ability to transduce a broad range of cell types (Table 3) [24,29,30,31,32,33,34,35,36,37]. It has been shown that transgenes continue to be expressed at the same level as at the time of administration and exhibit a dose-dependent effect [25,38]. Moreover, AAV-based therapy is considered safe since this vector often does not integrate into the host genome and remains in episomal form [26].

With the growing popularity of gene therapy and the increasing number of related studies, the issue of scaling up vector production arises. During scaling, many challenges are taken into consideration, such as a loss of functional viral titers during filtration and purification processes [39]. However, increasing titers during the production stage can partially lessen the losses at subsequent production stages. In the case of production using adherent cultures, scaling up involves increasing the surface area for cell cultivation, which in practice is achieved by adding parallel culture plates [40]. In turn, enhancing the process of viral particle production can also help minimize economic costs. Additionally, the need for higher AAV titers arises during clinical and preclinical studies, since they require large-scale production with significantly higher vector particle yields, compared to in vitro experiments [41].

Accordingly, there is a pressing need to obtain high AAV titers, driven by the growing scientific interest in this virus as a therapeutic agent and the necessity of scaling up its production. This review examines various approaches to increasing viral titers by targeting different stages of the production process.

## 2. Characteristics of AAV as a Vector

AAV is a small, single-stranded DNA virus belonging to the *Parvoviridae* family [42]. The virus has an icosahedral capsid with a mass of approximately 3.8 megadaltons and a diameter of about 260 Å. The capsid is composed of 60 subunits, represented by three proteins: VP1, VP2, and VP3. The AAV genome, approximately 4.7 kb in size, is surrounded by two inverted terminal repeats (ITRs), each about 145 nucleotides long [41].

AAV enters the cell via receptor-mediated endocytosis through the plasma membrane. Various proteoglycans, specific to different serotypes, act as primary receptors, for instance, the receptor for AAV serotype 2 is the heparan sulfate proteoglycan. In addition to these glycan interactions, binding to other surface proteins is also needed [42].

Once inside the cell, AAV generally remains as an episome and only rarely integrates into the host genome—an aspect that has drawn considerable attention in the scientific community [43]. Wild-type AAV has been shown to integrate into human chromosome 19 at the AAVS1 site, which contains motifs also present in the AAV ITR regions. Consequently, portions of the viral genome can integrate into host DNA through homologous recombination [44]. Research on mice shows that the majority of viral genome integration during therapy occurs within the T-cell receptor gene loci, which contain homologous regions enabling recombination processes [45].

Research on recombinant AAV integration into human hepatocytes has reported integration rates ranging from 0.6–2.8%, both in vivo and ex vivo. It was also observed that most integrated AAV sequences were significantly rearranged and accompanied by deletions of the genomic sequence at the integration site. Additionally, a dose-dependent effect has been observed, with higher viral inputs correlating with more integration events [26]. Furthermore, it was shown that a decrease in the level of DNA methylation in cells increases the frequency of AAV integration [46], and the presence of double-strand breaks increases the likelihood of viral integration [47]. In the context of CRISPR-Cas9 therapy, AAV vectors used to deliver the editing machinery demonstrated high integration frequencies [48]. Despite current data showing that in normal conditions, AAV integration rates remain below 3% [26], this makes it one of the safest vectors in terms of insertional mutagenesis when compared to adenoviruses or lentiviruses, which actively integrate into the host genome.

**Figure 1:**
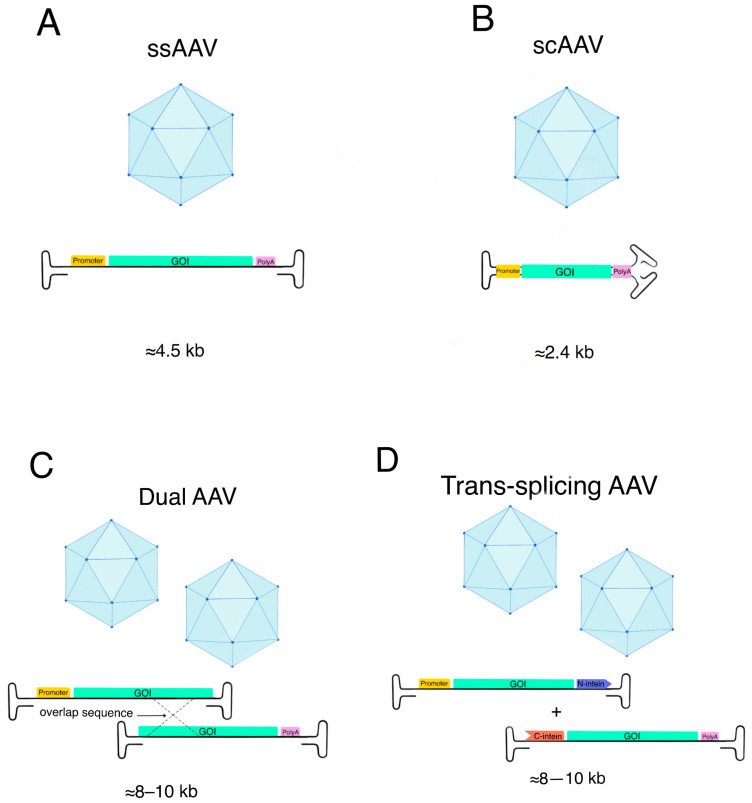
AAV vector strategy of delivery. (**A**) Single-stranded AAV, promoter region (orange) gene of interest (turquoise), and polyadenylation signal (purple). (**B**) Self-complementary AAV, promoter region (orange), gene of interest (turquoise), and polyadenylation signal (purple). (**C**) Dual AAV vector, promoter region (orange), gene of interest (turquoise), and polyadenylation signal (purple). (**D**) Trans-splicing AAV, promoter region (orange), gene of interest (turquoise), N-terminal fragment of the intein (blue), C-terminal fragment of the intein (red), and polyadenylation signal (purple).

When using AAV as a vector for gene therapy, it is essential to consider the size of the genome it can accommodate. A study investigating the effect of the genome size on viral particle production found that genomes of 1.9 kb and 3.4 kb resulted in similar yields of purified viral particles. However, a 4.9 kb genome produced four times fewer purified particles. Additionally, the proportion of empty capsids exceeded 50% for the 4.9 kb genome, compared to less than 33% for the smaller genomes [49]. Based on these findings, the genome size should be less than 4.9 kb, which represents one of the key limitations of AAV vectors in gene therapy. To overcome this constraint, modified vector designs have been developed. Self-complementary AAV (scAAV) delivers a double-stranded DNA genome, enabling faster and more efficient transgene expression by bypassing the need for second-strand synthesis; however, this approach further reduces the effective packaging capacity to approximately 2.4 kb (Figure 1B) [50,51]. In contrast, dual AAV vectors address the opposite challenge by splitting larger transgenes between two separate vectors, allowing for the delivery of constructs up to 8–10 kb via recombination or trans-splicing within target cells (Figure 1) [52,53].

Currently, the most pressing issue with AAV as a delivery vector is the immune response. In recent trials using next-generation AAV vectors, administered systemically at high doses, multiple patient fatalities have been reported—review articles estimate ~16 deaths attributed to immunotoxicity and organ failure following the intravenous administration of AAV at doses typically above 1 × 10^14^ vg/kg [54,55]. These tragic events underscore the risk of innate and adaptive immune activation, leading to liver, kidney, heart, or lung failure, especially in subjects with underlying comorbidities or pre-existing organ dysfunction. Two detailed case reports illustrate this risk. High-dose therapy with AAV serotype 9 (1 × 10^14^ vg/kg) resulted in the death of a 27-year-old patient with Duchenne muscular dystrophy. An innate immune response triggered acute respiratory distress syndrome and cardiac arrest 6 days after treatment with the transgene [56]. Another fatality occurred in a 16-year-old DMD patient treated with fordadistrogene movaparvovec at an even higher dose (2 × 10^14^ vg/kg), likely due to an innate immune response targeting the myocardium and resulting in cardiogenic shock [55,57,58]. These examples highlight the urgent need for dose optimization, immune modulation strategies, and the development of safer vector platforms. Current research shows that innate immunity against AAV is activated via Toll-like receptors (TLRs). Intracellular TLR9 contributes to the immune response to the transgene and capsid. In addition, inserted transgenes, promoter sequences, and RNA expressed by the vector may contain inflammatory signals recognized by TLRs [59]. After transduction, some AAV virions undergo ubiquitination and degradation in proteasomes, and their capsid peptides bind to MHC class I molecules, which are transported to the cell surface for antigen presentation, leading to the destruction of transduced cells by cytotoxic T cells [60]. Furthermore, in some young patients, high doses of AAV, equal to or exceeding 5 × 10^13^ vp/kg, led to thrombotic microangiopathy with hemolytic anemia, low platelet counts, and hemolytic–uremic syndrome, resulting in kidney damage, even when vectors were administered with steroids. The use of AAV vectors for the correction of spinal muscular atrophy in over 1400 individuals resulted in 9 cases of thrombotic microangiopathy in girls aged 4 months to 4 years [28]. In addition to the above mechanisms, the human body contains antibodies specific to different serotypes that neutralize AAV transduction. Population studies have shown that in the United States, the prevalence of antibodies to AAV1 ranged from 32% in Wisconsin to 67% in South Carolina, and in Europe, 48% of people in Sweden have neutralizing antibodies to AAV1, while in Poland and Hungary, this figure is 79%. Moreover, antibodies may exhibit cross-reactivity [27]. It is also important to note that the studies mentioned above report a dose-dependent effect, with higher virus concentrations leading to a more severe immune response [27,28,59]. In conclusion, there are suggestions, at the moment, for overcoming the immune response, such as using EDTA to block complement activation, developing new serotypes, and other methods to reduce antibody levels and evade them, including the use of various immunosuppressants and changes in administration methods and routes [61,62,63,64].

## 3. The Application of AAV-Based Therapeutics

Currently, AAV-based therapeutics are gaining significant popularity. The table below provides an overview of the latest gene therapy drugs based on AAV, the research on which began in 2024 (Table 4).

AAV is widely used in gene therapy. The main approach is gene replacement therapy, where a functional copy of a defective gene is introduced into living cells. Another strategy is genome editing, which can be enabled by CRISPR-based technologies [37].

In addition to treating congenital genetic defects, AAV-based therapy can be applied to stroke. Ischemic events in stroke lead to changes in the expression of a group of endogenous genes, and the use of AAV-mediated delivery could modulate gene expression, potentially aiding in the restoration of nervous system functions [65]. Experimental research has shown that AAV therapy in a rat model of ischemic stroke—induced by middle cerebral artery occlusion using an intraluminal suture—exerted beneficial effects. Following treatment, female rats exhibited reduced neuroinflammation and improved behavioral outcomes [66]. Another study showed that the downregulation of circulating forkhead box protein P1 (circFOXP1) in stroke accelerated the degradation of the signal transducer and activator of transcription 3 (STAT3) by binding to and enhancing its ubiquitination. This process ultimately worsened brain injury after cerebral ischemia by activating apoptotic pathways. The AAV-mediated overexpression of circFOXP1 significantly reduced apoptosis and improved functional recovery [67]. Another research group investigated the use of gene therapy for ischemic stroke. Granulocyte colony-stimulating factor (G-CSF) is a hematopoietic growth factor approved by the FDA for treating neutropenia. G-CSF stimulates the growth and differentiation of hematopoietic stem cells, and both G-CSF and its receptor are expressed in the central nervous system (CNS). However, the clinical use of hG-CSF is limited by its short half-life. Mice with bilateral common carotid artery occlusion received the AAV-mediated delivery of hG-CSF. The therapy reduced levels of C/EBP homologous protein (CHOP), glucose-regulated protein 78 (GRP78), dynamin-related protein 1 (DRP1), Beclin 1, p62, and LC3-phosphatidylethanolamine conjugate (LC3-II), improving the functional status of the mice with bilateral carotid artery occlusion [68].

Moreover, the idea of using AAV for treating cancer is currently being explored. A synthetic AAV, Ark313, has shown high transduction efficiency in mouse T-cells. The advantage of this method is its ability to deliver DNA without nucleofection, CRISPR/Cas9-mediated knockouts, or the targeted integration of large transgenes. Additionally, CAR-T cells generated using Ark313 showed enhanced efficacy in treating highly aggressive solid tumor Lewis carcinoma [69]. Another study showed that using an AAV vector to deliver tumor necrosis factor superfamily protein 14 (LIGHT) to endothelial cells in the brain during glioma immunotherapy prolonged survival. It also promoted T-cell anti-tumor responses by inducing the formation of tumor-associated high endothelial venules and tertiary lymphoid structures [70]. A recent study also demonstrated the effectiveness of AAV in treating glioblastoma. AAV targeting the HER2 receptor effectively transduced tumor cells with PD-1 immunoadhesin, inducing aPD-1 secretion into the tumor microenvironment, which enabled the reactivation of T-cells. Such combined therapy with AAV and anti-HER2.CAR/NK-92 cells can enhance the efficacy and minimize the side effects of glioblastoma immunotherapy [71]. The above data demonstrate a broader scope of application of AAV than a therapeutic agent.

## 4. AAV Production—Current Approaches to Increasing Titers

**Figure 2:**
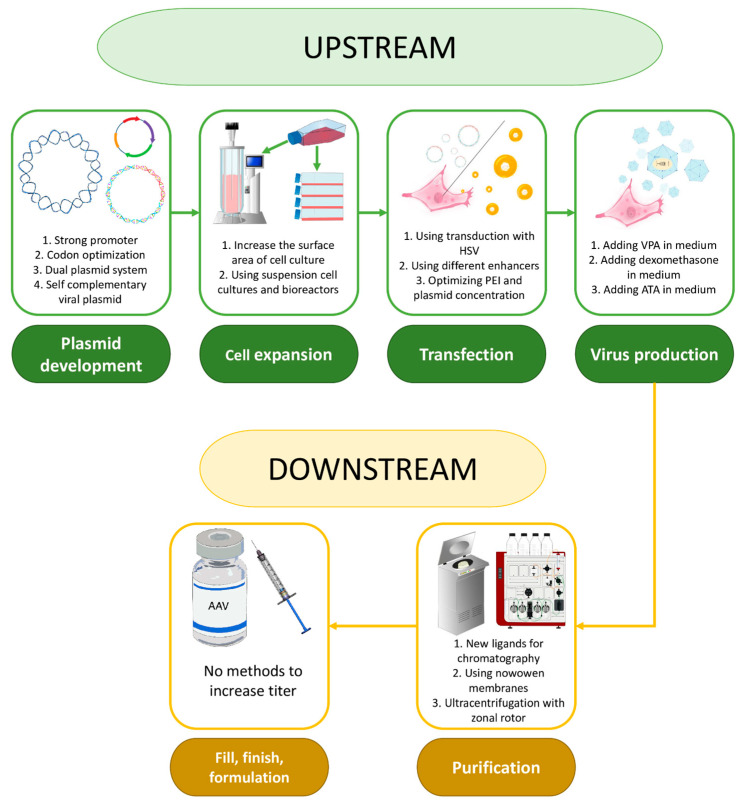
Stages of AAV-based drug development and approaches to increasing vector production at each stage.

The process of preparing viral vectors can be divided into three major stages, each of which is subdivided into smaller ones. The first stage, referred to as primary development, involves the plasmid design, expansion of producer cell cultures for viral particle production, plasmid transfection into these cells, and the production of viral particles. The next stage, secondary development, includes virus purification and concentration. The final stage involves preparing the virus in a form suitable for patient administration (Figure 2) [40]. In terms of increasing titers, the final stage is of limited relevance as it does not affect the number of viral particles already produced.

### 4.1. Approaches to Increasing Titers During Plasmid Development

In terms of improving the yield of viral particles, modifications can be made at the earliest stages of production. Typically, cells are transfected with three plasmids: (1) a helper plasmid (pHelper), which carries adenoviral serotype 5 genes necessary for viral assembly; (2) a Rep-Cap plasmid, which encodes the vector capsid proteins and replication machinery; and (3) a plasmid containing the target gene, also known as the vector plasmid [72,73]. Studies have suggested that functional AAV titers can be increased by transitioning from triple to dual plasmid transfection. This strategy involves incorporating the target gene into the Rep-Cap plasmid. This dual system increases the vector genome yield by 2.5-fold compared to triple-plasmid transfection. The reason is that the probability of co-transfecting two plasmids is higher than that of successfully delivering three plasmids in equal ratios into the same cell. This is critical, as the assembly of a functional viral particle requires the simultaneous presence of all essential genes, which in the triple-transfection system are distributed among three plasmids [74]. Additionally, other plasmid modifications have been developed. For instance, researchers created a self-complementary proviral plasmid, which reduced the cross-packaging of viral genomes (which can account for up to 13% of total particles in some studies). This improvement reduced the toxicity and led to a slight increase in functional titers [50].

### 4.2. AAV Producer Cell Lines

Currently, various producer cell lines are used for AAV production, with the most common being HEK 293 and its derivatives, CHO, V27, Sf9, and HeLa. Various modifications to these cell lines have been explored to enhance AAV yields. HEK 293 cells, which are derived from human embryonic kidney cells, were modified with a gene encoding the viral protein SV40 Large T-antigen to improve transfection efficiency, and were later designated as HEK293T [75]. Further genomic modifications of HEK cells have also been shown to enhance AAV production. For instance, one study used the CRISPR-Cas9 system to overexpress genes involved in endoplasmic reticulum protein processing and anti-apoptotic pathways, resulting in significant titer improvements. The overexpression of BCL2 increased productivity by 79%, HSPA6 by 54%, GADD34 by 71%, and XBP1 by 48% [76]. In another study, helper genes E2A and 22K/33K—typically present in helper plasmids used during triple transfection—were stably integrated into HEK 293 cells. This modification reduced the requirement to only capsid and target gene plasmid transfection, with efficiency comparable to that of transient transfection in standard HEK 293 cells [77]. A similar approach was applied to integrate capsid protein genes into producer cells, eliminating the need for triple co-transfection. For AAV production, these modified cells require transfection with only the plasmid containing the target gene. A stable HEK 293-derived cell line implementing this strategy was developed as early as 2002 [78].

A recent study developed stable AAV8 producer cell lines based on HEK 293 cells. These rAAV8-producing lines were generated through stepwise transfection with synthetic genetic modules cloned into transposon vectors. In the first stage, three constructs were introduced: (1) the Genomic Module (GM), which contained an inducible GFP cassette under the control of LacSwitch, flanked by AAV2 ITRs, as well as the lacI gene linked to a puromycin resistance marker; (2) the Replication Module (RM), which included inducible elements of the adenoviral helper (E4orf6), Rep68-mCherry, DNA-binding protein (DBP), and Tet-rtTA3, along with a hygromycin resistance marker; and (3) the Packaging Module PM8-B, which encoded AAV8 cap (with a non-functional start codon for VP1), Rep52 and smURFP, as well as the CymR repressor with a blasticidin resistance marker. These vectors were integrated using Transposase 1, and after antibiotic selection, a pool of VH cells was isolated. At the second stage, VH1 and VH3 cells were re-transfected with one of two capsid modules (CM8-A or CM8-B), containing the capsid gene VP123 (±Rep52) and TagBFP, using Transposase 2. After FACS sorting and productivity assessment, clones VH3B1 and VH3B2 were selected as stable rAAV8 producer cell lines [79]. The efficiency of rAAV8 production in VH3B1 and VH3B2 was comparable to that of the conventional triple transfection method [79].

Another study published in 2023 described the development of an AAV producer line based on HEK 293 cells that demonstrated infectious rAAV titers comparable to the conventional adenoviral helper method. Stable constructs encoding AAV Rep68, adenoviral E4orf6, and DBP were integrated into HEK 293 cells. Expression was controlled by mifepristone-inducible promoters. Rep68 carried a ligand-sensitive destabilization domain to enable regulated expression. The resulting line, RM4, enabled the enhanced transcription and replication of rAAV upon induction, eliminating the need for adenoviral co-infection [80].

CHO cells (Chinese hamster ovary cells) are another option for AAV production. Despite their ability to produce AAV, the use of CHO cells is complicated by limited cap gene expression, necessitating the inclusion of additional auxiliary plasmids alongside the standard three-plasmid system [81]. A potential solution to this problem involves the use of herpesvirus-based transduction systems. Specifically, in a 2023 study, CHO cells were modified to express herpesvirus entry mediator proteins (HVEM and/or Nectin-1), which are required for herpesvirus infection. Among these, CHO cells expressing only HVEM were shown to be the most effective in producing rAAV9 particles [82].

Sf9 cells, which are epithelial cells derived from the ovarian tissue of fall armyworm (Spodoptera frugiperda) pupae, represent another alternative for AAV production. These cells offer several advantages over HEK 293 cells, including higher AAV yields, a lower empty capsid content, reduced viral particle aggregation, and greater stability at concentrations exceeding 10^13^ vg/mL [83]. The OneBac2.0 system, based on baculovirus, was developed to enable vector assembly upon the infection of Sf9 producer cells. Compared to HEK 293-produced AAV, the OneBac2.0 system showed the reduced packaging of foreign DNA due to the removal of the Rep-binding site from rep/cap expression constructs. Moreover, although AAV particles can carry antibiotic resistance genes, those produced using OneBac2.0 contained fewer plasmid-derived ampicillin resistance sequences [84,85]. One of the drawbacks identified in a 2022 study was the presence of a higher proportion of unresolved genomes, which correlated with the formation of truncated, self-complementary, single-stranded ITR species in Sf9-based systems, as compared to HEK 293 cells [86].

In the same year, a stable insect cell line based on Sf9 was developed for rAAV packaging by integrating the Rep gene in the form of two separate cassettes: Rep78 expression was driven by the 39k (or ΔIE-1) promoter, while Rep52 was under the control of the polH promoter, ensuring the temporal coordination of protein synthesis. To enhance transcription, the constructs included baculoviral enhancers hr2.09, hr4b, and hr5, which regulate expression depending on the stage of infection and the promoter type. The genetic construct also contained a modified Cap cassette with an artificial intron inserted into the capsid gene and an ACG start codon at the VP1 position, allowing the fine-tuning of VP1, VP2, and VP3 protein ratios. To reduce basal expression and increase stability, non-overlapping regulatory elements and lowered promoter activity prior to recombinant baculovirus induction were employed. This construct architecture enabled high and stable rAAV production without significant titer reduction over at least nine passages [87].

HeLa cells represent yet another system for AAV production. HeLa-based systems are versatile and allow for the straightforward generation of new vectors and transgene constructs [88]. However, these systems require the stable amplification of rep and cap genes, which is achieved either by transfection or adenovirus-mediated transduction [89]. Despite their advantages, HeLa cells are rarely used today and have largely been replaced by more efficient producer cell lines.

However, in 2011, a universal method for generating stable cell lines, including those based on HeLa cells, was proposed. Stable HeLa and A549 cell lines were developed by integrating cassettes carrying the rep2 and cap genes of the selected serotype, along with a transgene cassette flanked by ITRs, into the genome, enabling complete AAV assembly within a single cell. Gene expression was induced upon infection with a helper adenovirus (Ad5 or Ad/AAV hybrid), which triggered rep and cap amplification and initiated vector assembly. The most productive line, A549-K209, demonstrated up to a 1000-fold amplification of rep/cap copies and required 5–10 times less adenovirus compared to the HeLa-derived clone B50, which was also used in this study. Productivity reached 6.4 × 10^12^ gc per 10^9^ cells, and stability was maintained for over 60 population doublings, with more than 70% of the produced capsids containing full genomes. This system eliminates the need for transfection and enables the creation of an inducible, stable AAV producer cell line [90].

A modern approach to scaling up AAV production involves replacing adherent cultures with suspension cultures, which can be grown in bioreactors, thereby increasing the producer cell density without requiring an additional surface area. Moreover, several studies have reported that adherent HEK 293 cells can be adapted to the suspension culture without the use of animal-derived components or antibiotics [91]. AAV vectors produced in suspension cultures are bioequivalent to those derived from adherent cultures and may even exhibit higher efficacy [92]. However, a significant drawback of suspension cultures is their lower transfection efficiency [93].

To date, seven AAV-based therapeutics have been approved by the FDA. These include products derived from transient expression systems based on HEK 293 cells (Luxturna, Zolgensma, Elevidys, Beqvez, and Kebilidi) and products of the Sf9/baculovirus system (Hemgenix, Roctavian) [88]. The transient transfection system remains the simplest and most flexible approach and is, therefore, widely used in AAV vector development. It can be scaled up to several thousand liters, making it attractive in resource-limited settings. A more scalable alternative is the baculovirus system, which allows for high yields, but may potentially reduce the in vivo vector potency due to differences in post-translational modifications [88].

### 4.3. Approaches to Increasing Titer During Transfection

Enhancing the AAV yield can be achieved by improving and optimizing the transfection process. However, there is evidence that with a transfection efficiency of 60%, only about 7% of cells in a triple transfection model produce measurable amounts of assembled AAV capsids. Interestingly, co-transfection with an infectious AAV2 clone and a helper plasmid increases the proportion of cells positive for assembled AAV capsids by 4–5 times [94]. One approach to improving this step involves replacing plasmid-based transfection with the use of herpes simplex virus (HSV). AAV producer cell lines were infected with HSV at a multiplicity of infection (MOI) of 12. The producer clones generated approximately 1.6 × 10^4^ AAV9 virions per cell. These results demonstrate the higher efficiency of this method compared to plasmid-based transfection, along with its scalability and equal effectiveness in both adherent and suspension cultures [95].

Nevertheless, despite the limitations of plasmid-based transfection, there may be situations in which replacing this method with the alternatives mentioned above is not feasible. In such cases, optimizing the existing process becomes critical. In 2018, patent WO2018226887A1 was filed, describing compositions and methods for the high-efficiency transfection of cells with molecules such as nucleic acids (e.g., plasmids). The authors reported a 10-fold increase in the final titer. The proposed improvements included the use of various enhancers and modifications to the polyethyleneimine (PEI) to DNA ratio. Depending on the cultivation conditions, different PEI-to-DNA ratios demonstrated varying levels of efficiency. Notably, regardless of the cultivation conditions, the most effective PEI-to-DNA ratios were found to be 2:1, 2.5:1, and 3:1.

In addition to improving the transfection process, there are methods for modifying the AAV genome to enhance viral particle production. The transcription of the rep gene is initiated from the p5 or p19 promoters, which encode the large (Rep78 and Rep68) and small (Rep52 and Rep40) nonstructural Rep proteins, respectively [96]. The two large Rep proteins (Rep78 and Rep68) specifically bind to DNA and cleave duplex ITRs in a site- and strand-dependent manner. This cleavage occurs at the terminal resolution site (trs) within the ITR and enables the replication of the linear genome ends [97]. The modification of the ITR region by removing the *trs* sequence prevents cleavage by Rep68/78, resulting in the duplication of the vector genome joined by a modified internal ITR. The delivery of the duplicated genome to the nucleus facilitates self-repair and the formation of a double-stranded DNA structure without the need for complementary strand synthesis. As a result, the transcription of the gene of interest and subsequent expression of its encoded protein begin earlier and are maintained at higher levels [41].

### 4.4. Approaches to Increasing Titer During Purification and Concentration

The next stage in AAV production, which causes a significant loss of functional titer and requires optimization, is filtration and purification [39]. A study by Mikako Wada and colleagues developed a rapid, large-scale method for AAV purification using two-step CsCl gradient ultracentrifugation with a zonal rotor. This method allows for the quick and efficient separation of full-genome AAV particles from empty ones. The presence of highly purified AAV particles with full genomes was verified through analytical ultracentrifugation, digital PCR, transduction efficiency assays, and transmission electron microscopy (TEM). To remove CsCl from the purified AAV vector fractions, a polishing method was developed using hydroxyapatite column chromatography. A modification of this method involving the addition of CaCl_2_ increased the AAV recovery efficiency to 85%. This approach was tested across multiple AAV serotypes, suggesting its broad applicability [98].

In addition to ultracentrifugation, fiber-based nonwoven membranes can be used for AAV purification. The developed anion-exchange membrane AEX-TEA demonstrated a high binding capacity for AAV2 (9.6 × 10^13^ capsids/mL) and productivity (2.4 × 10^13^ capsids/mL·min), while achieving a logarithmic host protein recovery value of 1.8. Under identical loading and operational conditions, the commercial Sartobind Q membrane binds 1.9 × 10^13^ capsids/mL with a similar logarithmic recovery value of 1.8 for host cell proteins. These data highlight the effectiveness of nonwoven membranes in extracting the virus from the culture medium [99].

Another approach to improving the purification process involves enhancing chromatography methods. New peptide ligands for serotype-independent affinity chromatography have been developed. For instance, the peptide ligand A10 demonstrated a high binding capacity (over 10^14^ virions per milliliter of resin) and purification efficiency across all AAV serotypes. Additionally, alkaline-resistant variants of A10 were introduced, capable of performing multiple purification cycles for AAV2, AAV8, and AAV9 with intermediate alkaline cleaning, without a loss of efficiency [100]. The purification methods described above offer new strategies for improving the process efficiency and reducing AAV functional titer loss.

These studies present modern methods to address the problem of the titer loss and low yield of viral particles at various stages of AAV production. However, most of these approaches are costly, requiring extensive process optimization and significant time and effort, which remain major drawbacks.

## 5. AAV Vector Production

In addition to the previously described approaches, it has been suggested that using various anti-inflammatory agents at low concentrations may enhance protein production and, consequently, capsid formation for AAV vectors [101].

In 2006, while attempting to inhibit the early stage of the vaccinia virus with aurintricarboxylic acid (ATA), it was discovered that adding ATA to AD293 cells increased adenovirus 5 production [102]. A subsequent study examined the effects of ATA on HSV-1 production in V27 cells, demonstrating that ATA significantly increased viral particle production and reduced the cytopathic effect by 20–60%. Similar experiments on other strains and cell cultures showed no adverse effects from ATA, even in cases where no increase in viral particle production occurred, although McIntyre strain production in HEK293 cells also increased significantly. It was also noted that ATA’s activity was independent of serum presence in the medium [101].

Other compounds at low concentrations can similarly enhance viral particle yields. One such compound is valproic acid (VPA), which can inhibit the induction of several interferon-sensitive antiviral genes, thereby increasing the transcription of viral genes and improving viral spread [103]. Various studies have investigated the effectiveness of adding VPA during cell culturing to increase protein production. For instance, the effect of VPA on enhancing antibody production by CHO cells was studied. While VPA increases the protein yield, it also inhibits cell growth, which was identified as a disadvantage [104]. However, since viral vector production often relies on plasmid transfection, the episomal localization of plasmids in the nucleus can lead to their dilution during cell division. Therefore, limiting cell division could potentially enhance viral particle yields.

Moreover, a team of researchers filed the previously mentioned patent application WO2018226887A1 in an effort to increase the viral particle yield. In this patent, they described not only methods to enhance transfection, but also the use of VPA to boost AAV titers. The use of VPA in optimized PEI-mediated transfection increased AAV vector titers by approximately tenfold compared to production without enhancers. This method was effective in both adherent cultures and bioreactor-based production. It is worth noting that in this study, VPA was applied during transfection, making it difficult to determine whether the increased vector yield was due to improved transfection efficiency or, as in the cases described above, enhanced protein production by host cells. The authors also mentioned the possibility of using VPA salts, such as sodium valproate and potassium valproate.

A similar patent application (US20190290710A1) was filed in 2017, describing the use of glucocorticoid analogs to enhance AAV vector yields. Dexamethasone was the primary compound used, with the most effective concentration being 0.1 μM, which increased AAV production in HeLa S3 cells. Notably, dexamethasone enhanced both extracellular and total AAV yields at a concentration as low as 0.01 μM. The method was also shown to be effective in both adherent and suspension cultures, making it easily scalable.

A recent study investigated the effects of several low-molecular-weight compounds on increasing AAV titers. While VPA did not show significant efficacy, other compounds were effective. Nocodazole at 4 μM increased the AAV yield by 1.34-fold, while M334, a synthetic analog of trichostatin A (a class I and IIB histone deacetylase inhibitor), increased the yield by 1.36-fold at 2.5 μM. The caspase inhibitor z-VAD-fmk showed no significant efficacy. Notably, adding nocodazole 4 h after transfection further increased its effectiveness, raising the AAV titer by 2.2-fold. Combining nocodazole and M334, added 4 h post-transfection, increased the AAV titer by 2.6-fold compared to the control. The combined use of nocodazole and z-VAD-fmk improved cell viability, but reduced viral titers [105].

To summarize, the described methods offer significant advantages. They are readily applicable to almost any protocol, as they target a stage of production that typically undergoes the least optimization. All these techniques require neither advanced nor expensive equipment, are easy to implement, and can be scaled up without major difficulties. The proposed compounds are safe for human use and have either been approved by the FDA or are utilized in scientific research for the treatment of humans. Dexamethasone, both in its pure form and as part of various formulations, is included in the FDA-approved list. In addition to dexamethasone itself, derivatives such as dexamethasone acetate and dexamethasone sodium phosphate have also been approved. Valproic acid and its sodium salt are similarly listed among FDA-approved drugs. However, aurintricarboxylic acid and its derivatives have not yet received FDA approval, but are being actively studied as antiviral agents. Nocodazole is also not FDA-approved for the treatment of human diseases and is currently used exclusively in scientific research.

## 6. Conclusions

In recent years, interest in gene therapy and AAV as vectors for delivering therapeutic genetic constructs has grown significantly. Over half of clinical trials for novel treatments targeting inherited diseases are based on AAV-mediated therapy. Moreover, successful attempts have been made to use AAV therapy for cancer, ischemic heart disease, stroke, and other conditions. However, side effects of AAV therapy—previously underestimated—are now increasingly recognized, including a higher-than-expected probability of genome integration and immune responses to the vector, especially at high doses or with repeated administrations. Nonetheless, potential solutions to these challenges are already being developed, and together with the expanding number of clinical and preclinical studies, this opens promising prospects for overcoming current limitations.

One of the main challenges remains the large-scale production of AAV vectors. Key limitations include a low viral particle yield, scalability issues with adherent cell cultures, high production costs, and variability in the transduction efficiency. In response, several breakthrough strategies have been introduced, such as optimizing triple transfection systems, employing suspension-adapted producer cell lines, improving vector genome designs (e.g., self-complementary, dual AAV systems, and trans-splicing AAV systems), and refining downstream purification processes. Additionally, the use of small molecules like valproic acid, dexamethasone, aurintricarboxylic acid, and nocodazole has demonstrated increased AAV productivity. Another promising approach involves helper viruses such as herpes simplex virus (HSV) to deliver the AAV genome into producer cells, offering a potentially more scalable and consistent alternative to plasmid-based transfection.

Despite this progress and the emergence of promising solutions, further research is still needed to establish the most effective strategies for the large-scale development and production of AAV-based therapeutics. Achieving this will unlock the vast potential of AAV vectors for treating a wide range of diseases in the future.

## Figures and Tables

**Table 1:** FDA-approved AAV-based gene therapy.

Generic Name	Brand Name	Company	Date of Approval	Serotype	Gene	Disease
Voretigene neparvovec-rzyl	LUXTURNA	Spark Therapeutics, Inc.	19 December 2017	AAV2	RPE65	Leber congenital amaurosis
Onasemnogene abeparvovec-xioi	ZOLGENSMA	Novartis Gene Therapies, Inc.	24 May 2019	AAV9	SMN1	Spinal muscular atrophy
Etranacogene dezaparvovec-drlb	HEMGENIX	CSL Behring LLC	22 November 2022	AAV5	Factor IX	Hemophilia B
Valoctocogene roxaparvovec-rvox	ROCTAVIAN	BioMarin Pharmaceutical Inc.	29 June 2023	AAV5	Factor VIII	Hemophilia A
Fidanacogene elaparvovec-dzkt	BEQVEZ *	Pfizer, Inc.	26 April 2024	AAVrh74var	Factor IX	Hemophilia B
Delandistrogene moxeparvovec-rokl	ELEVIDYS	Sarepta Therapeutics, Inc.	20 June 2024	AAVrh74	Micro-dystrophin	Duchenne muscular dystrophy
Eladocagene exuparvovec-tneq	KEBILIDI (Upstaza)	PTC Therapeutics	13 November 2024	AAV2	DDC	Aromatic L amino acid decarboxylase (AADC) deficiency

* Development and commercialization have been globally discontinued by Pfizer as of February 2025 due to weak demand from patients and physicians.

**Table 2:** Viral delivery systems.

Vector	Packaging Capacity	Genome Type	Advantages	Limitations
γ Retroviruses	8 kb	ssRNA	1. Capable of stable expression [11]. 2. Relatively large genome capacity.	1. Capable of infecting only dividing cells [12]. 2. Integration into the genome primarily in the transcription initiation region [12]. 3. Retrovirus integration can lead to insertional mutagenesis, causing genotoxicity in cells. [13].
Lentiviruses	8 kb	ssRNA	1. Capable of transducing both dividing and non-dividing cells efficiently [14]. 2. Can be modified for selective delivery to lymphocytes [15]. 3. Relatively large genome capacity.	1. Integration into the genome, often near oncogenes, leading to insertional mutagenesis and the development of lymphomas [12]. 2. Provoking an immune response, which also limits transduction [16].
Adenoviruses	<7.5 kb (1st gen.) <14 kb (2nd gen.)	dsDNA	1. High-capacity adenoviral vectors can transfer a genome up to 37 kb [17]. 2. Do not integrate into the genome, remain as episomes, and efficiently transduce cells [16]. 3. Transduce both dividing and non-dividing cells [18].	1. Inducing strong immune response and severe inflammatory processes [16]. 2. Many people have pre-existing immunity to adenoviruses, with up to 90% immunity to certain serotypes. [19].
Herpesviruses	>30 kb	dsDNA	1. Capable of carrying very large and multiple transgenes [20]. 2. Capable of infecting host cells without integration into the genome [20]. 3. HSV-1 has tropism for certain neuronal cells [21].	1. The smallest number of studies and publications on the use of this virus, as a vector for gene therapy, compared to other delivery systems. 2. Some virus strains may exhibit toxicity [22].
AAVs	~4.5 kb	ssDNA	1. Capable of transducing both resting and dividing cells [23]. 2. Different serotypes exhibit tissue- and organ-specificity [24]. 3. The vector remains as an episome, without causing insertional mutagenesis [25]. 4. Express transgenes at levels similar to those at the time of administration [26]. 5. Have the highest number of publications and research, making it the most extensively studied.	1. Relatively small insertion size. 2. Immunogenic upon repeated administration or high-dose therapy [27,28].

**Table 3:** AAV serotype tropism.

Serotype	Primary Receptors	Co-Receptors	Tissue Tropism in Humans
AAV1	N-linked sialic acid	AAV receptor (AAVR)	CNS, retina, pancreas, skeletal muscle
AAV2	heparan sulfate proteoglycan	FGFR1, LamR, CD9, Tetraspanin, αVβ5 and α5β1 integrins	Retina
AAV3	heparan sulfate proteoglycan	FGFR1, HGFR, LamR	Liver
AAV4	O-linked sialic acid	Unknown	CNS, Lung
AAV5	N-linked sialic acid	PDGFR	CNS, retina, kidney, pancreas, liver
AAV6	heparan sulfate proteoglycan, N-linked sialic acid	EGFR	Skeletal muscle
AAV7	Unknown	Unknown	Skeletal muscle
AAV8	Unknown	LamR	CNS, heart, liver, retina
AAV9	terminal N-linked galactose	putative integrin, LamR	CNS, heart

**Table 4:** Latest developments in AAV-based gene therapy drugs.

Drug Name	Disease	Clinical Trial Phase	Vector	Estimated Study Completion Date
JAG201, USA	Phelan-McDermid syndrome, SHANK3 haploinsufficiency	Phase 2	AAV9	2031-06
SNUG01, China	Amyotrophic lateral sclerosis (ALS)	Early phase 1	AAV9	2029-10-15
AAV5-hRKp.RPGR	X-linked pigmentary retinitis	Phase 2	AAV5	2030-10-24
BBM-D101, China	Duchenne muscular dystrophy	Early phase 1	rAAV *	2030-07-31
GT-UGT1A1-AAV8-02, Russia	Crigler-Najjar syndrome type I	Phase 2	AAV8	2029-11-01
FLT201, USA	Gaucher disease type 1	Phase 2	AAVS3	2029-05
EXG110, China	Fabry disease	-	rAAV *	2027-04-09
JWK008, China	Mucopolysaccharidosis type I	Phase 1	AAV5	2029-06-22
GC301, China	Pompe disease	Phase 2	AAV9	2026-12
FBX-101, USA	Krabbe disease	Observational study	AAVrh.10	2029-12
Delandistrogene Moxeparvovec, USA	Duchenne muscular dystrophy	Phase 1	rAAVrh74	2026-09-30
SCG0106, China	Diabetic macular edema	Phase 1	rAAV *	2026-01
TN-401, USA	Arrhythmogenic right ventricular cardiomyopathy (ARVC)	Phase 1	AAV9	2029-10-01
SRD-001, USA	Dilated cardiomyopathy associated with Duchenne muscular dystrophy	Phase 1	AAV1	2030-02
GS-100, USA	NGLY1 deficiency	Phase 2	AAV9	2028-01-31
SGT-003, USA	Duchenne muscular dystrophy	Phase 2	AAV-SLB101	2031-05-06
JWK-007, China	Duchenne muscular dystrophy	Phase 1	rAAVrh74	2028-12-31
BBM-H803, China	Hemophilia A	Phase 2	rAAV *	2030-06-30
AMT-162, USA	Amyotrophic lateral sclerosis (ALS)	Phase 2	rAAVrh10	2031-03-30
GSL222, USA	Hemophilia B	Phase 3	AAV5	2028-10
SCG0106, USA	Neovascular age-related macular degeneration	Phase 2	rAAV *	2026-01-30
GC304, China	Familial hypertriglyceridemia	Phase 1	AAV5	2028-12
ICM-203, Australia	Knee osteoarthritis	Phase 2	rAAV5.2	2026-11
AGTC-501, USA	X-linked pigmentary retinitis	Phase 3	AAV2	2029-10

* Specific AAV serotypes were not disclosed in publicly available clinical trial data.

## Abbreviations

The following abbreviations are used in this manuscript:

AAV	Adeno-associated virus
ATA	Aurintricarboxylic acid
aPD-1	anti-PD-1 immunoadhesin
BCL2	B-cell lymphoma 2
CAR-NK	Chimeric antigen receptor natural killer
CHOP	C/EBP homologous protein
DRP1	Dynamin-related protein 1
EGFR	Epidermal growth factor receptor
FGFR1	Fibroblast growth factor receptor 1
GADD34	Growth arrest and DNA damage-inducible protein
GRP78	Glucose-regulated protein 78
HER2	Human epidermal growth factor receptor 2
hG-CSF	Human granulocyte-colony-stimulating factor
HGFR	Hepatocyte growth factor receptor
HSPA6	Heat-shock protein family member 6
HVEM	Herpesvirus entry mediator
IFN	Interferon
LC3-II	LC3-phosphatidylethanolamine conjugate
LIDHT	Tumor necrosis factor superfamily protein 14
P62	Sequestosome-1
PD1	Programmed cell death 1
PDGFR	Platelet-derived growth factor receptor
PEI	Polyethyleneimine
TLR	Toll-like receptor
VPA	Valproic acid
z-VAD-fmk	carbobenzoxy-valyl-alanyl-aspartyl-[O-methyl]-fluoromethylketone
XBP1	X-box binding protein 1
